# Acute inhibition of NCC does not activate distal electrogenic Na^+^ reabsorption or kaliuresis

**DOI:** 10.1152/ajprenal.00339.2013

**Published:** 2014-01-08

**Authors:** Robert W. Hunter, Eilidh Craigie, Natalie Z. M. Homer, John J. Mullins, Matthew A. Bailey

**Affiliations:** ^1^University/British Heart Foundation Centre for Cardiovascular Science, University of Edinburgh, Edinburgh, United Kingdom; and; ^2^Centre for Nephrology, University College London Medical School, Royal Free Campus, London, United Kingdom

**Keywords:** NaCl cotransporter, epithelial Na^+^ channel, hydrochlorothiazide, benzamil

## Abstract

Na^+^ reabsorption from the distal renal tubule involves electroneutral and electrogenic pathways, with the latter promoting K^+^ excretion. The relative activities of these two pathways are tightly controlled, participating in the minute-to-minute regulation of systemic K^+^ balance. The pathways are interdependent: the activity of the NaCl cotransporter (NCC) in the distal convoluted tubule influences the activity of the epithelial Na^+^ channel (ENaC) downstream. This effect might be mediated by changes in distal Na^+^ delivery per se or by molecular and structural adaptations in the connecting tubule and collecting ducts. We hypothesized that acute inhibition of NCC activity would cause an immediate increase in Na^+^ flux through ENaC, with a concomitant increase in renal K^+^ excretion. We tested this using renal clearance methodology in anesthetized mice, by the administration of hydrochlorothiazide (HCTZ) and/or benzamil (BZM) to exert specific blockade of NCC and ENaC, respectively. Bolus HCTZ elicited a natriuresis that was sustained for up to 110 min; urinary K^+^ excretion was not affected. Furthermore, the magnitude of the natriuresis was no greater during concomitant BZM administration. This suggests that ENaC-mediated Na^+^ reabsorption was not normally limited by Na^+^ delivery, accounting for the absence of thiazide-induced kaliuresis. After dietary Na^+^ restriction, HCTZ elicited a kaliuresis, but the natiuretic effect of HCTZ was not enhanced by BZM. Our findings support a model in which inhibition of NCC activity does not increase Na^+^ reabsorption through ENaC solely by increasing distal Na^+^ delivery but rather by inducing a molecular and structural adaptation in downstream nephron segments.

systemic Na^+^ and K^+^ homeostasis is largely dependent on net solute transport in the distal renal tubule (35). Na^+^ is reabsorbed through the electroneutral NaCl cotransporter (NCC) in the distal convoluted tubule (DCT) and through the epithelial Na^+^ channel (ENaC) in the connecting tubule (CNT) and collecting ducts (CDs). The latter process is electrogenic, depolarizing the apical membrane and generating a transepithelial potential difference favoring the secretion of K^+^ into the tubular lumen through renal outer medullary K^+^ (ROMK) and large-conductance K^+^ (BK) channels. The balance between electroneutral and electrogenic Na^+^ reabsorption thus determines net renal K^+^ excretion, and this may predominate over any effect of changes in the expression of renal K^+^ channels (12, 35). This balance is therefore tightly regulated by intracellular signaling networks (notably the WNK-SPAK system) in response to systemic inputs including volume status, K^+^ status, and aldosterone (1, 18). That aldosterone is able to stimulate net Na^+^ reabsorption during volume depletion or K^+^ excretion during hyperkalemia has been termed the “aldosterone paradox.” Striking an appropriate balance between electroneutral and electrogenic Na^+^ transport in the distal renal tubule is key to resolving this paradox, and there is growing acceptance of a model in which the WNK kinase network forms a site at which cues to systemic volume and K^+^ status are integrated and translated into an effector response directed at fine tuning NCC, ENaC, and ROMK activities (1, 14).

The molecular details underlying these responses remain obscure, particularly those participating in minute-to-minute solute homeostasis. Early micropuncture and microcatheterization studies (7, 8, 44) in the rat revealed that the pathways for Na^+^ reabsorption in the CD ordinarily operate well below their saturation point, so that Na^+^ reabsorption in the CD is load dependent. Thus, suppression of NCC activity will result in the increased delivery of Na^+^ to the CNT and CDs, which will increase Na^+^ reabsorption through ENaC. Similarly, an increase in NaCl transport via NCC should result in a reduction in Na^+^ reabsorption through ENaC. This effect is commonly cited as the cause of hypokalemia in Gitelman's syndrome (11, 46) and during therapy with thiazide diuretics (9, 52) and of hyperkalemia in Gordon's syndrome (27, 58). All of these states induce changes in the expression of ENaC and K^+^ channels and/or epithelial remodeling in the distal renal tubule (27, 29, 38, 58), often attributed to a neurohormonal adaptation to chronic perturbations in electrolyte and volume status (e.g., aldosteronism in Gitelman's syndrome). However, recent data have suggested that changes in NCC function may participate in the acute renal response to dietary K^+^ loading (48). We therefore hypothesized that inhibition of NCC activity would have an immediate effect on urinary K^+^ excretion by altering the distal delivery of Na^+^ and thus the net flux through ENaC. However, we found that the acute natriuretic effects of hydrochlorothiazide (HCTZ; NCC blockade) and benzamil (BZM; ENaC blockade) were additive and that urinary K^+^ excretion was not enhanced after a bolus of HCTZ. Our findings support a model in which acute changes in NCC activity per se do not contribute to the regulation of renal K^+^ excretion.

## MATERIALS AND METHODS

### 

#### Animals.

All experiments were conducted in accordance with United Kingdom Home Office regulations and the Animals (Scientific Procedures) Act of 1986. C57BL/6JOlaHsd wild-type mice were supplied by Harlan UK. Before experimentation, mice had free access to water and standard chow containing 0.25% Na^+^, 0.38% Cl^−^, and 0.67% K^+^. In a subset of experiments examining the response to dietary Na^+^ restriction, mice were maintained on chow containing 0.01% Na^+^ for 3 days. Mice were moved to a clean cage when the low-Na^+^ diet was introduced and again after 24 h. The baseline characteristics of the various experimental cohorts are shown in Table 1.

**Table 1.** Cohorts used for renal clearance

	*n*	Age, days	Body Weight, g
HCTZ time course (*clearance protocol A*)			
Vehicle control	5	76.4 ± 7.2	25.4 ± 2.3
HCTZ (2 mg/kg)*	9	79.1 ± 6.0	24.5 ± 0.8
BZM time course (*clearance protocol B*)			
Vehicle control	4	82.0 ± 1.2	25.8 ± 1.3
BZM (0.2 mg/kg)	4	84.5 ± 0.6	26.0 ± 1.8
BZM (2 mg/kg)	6	80.3 ± 2.9	26.2 ± 1.9
BZM (8 mg/kg)	5	82.8 ± 3.5	26.9 ± 1.4
HCTZ dose range (*clearance protocol C*)			
Vehicle controls†	9	75.4 ± 7.7	25.4 ± 2.1
HCTZ (0.2 mg/kg)	4	69.0 ± 4.2	24.3 ± 0.6
HCTZ (2 mg/kg)	11	82.2 ± 8.7	24.9 ± 1.4
HCTZ (20 mg/kg)	8	81.0 ± 16.6	24.6 ± 2.5
HCTZ (2 mg/kg) on 0.01% Na^+^ diet	6	90.3 ± 7.1	25.4 ± 2.5
HCTZ and BZM (*clearance protocol D*)			
BZM plus vehicle control	6	75.3 ± 3.8	24.7 ± 1.3
BZM plus HCTZ	6	75.2 ± 4.3	25.3 ± 2.5
BZM plus HCTZ on 0.01% Na^+^ diet	8	97.8 ± 8.6	27.6 ± 2.7

Data are means ± SD; *n*, no. of animals/group.

* The hydrochlorothiazide (HCTZ) cohort used to study the time course of the response (*clearance protocol A*) was also used to generate the data for nine animals in the 2 mg/kg group in the dose-range experiment, by pooling appropriately timed samples. †The vehicle control group in the dose-range experiment comprised the vehicle control group from the time-course experiment (1% DMSO in 0.9% NaCl, *n* = 5) plus additional mice who received 2% DMSO in 0.9% NaCl (*n* = 4) to match the vehicle used to carry HCTZ at 20 mg/kg. As there were no differences in the results obtained from these two vehicle groups, the data were pooled in the final analysis. BZM, benzamil.

#### Renal clearance.

Renal clearance experiments were performed as previously described (3). Mice were anesthetized with Inactin (thiobutabarbital sodium salt hydrate, Sigma), and catheters were inserted into the trachea, jugular vein, carotid artery, and bladder. A bolus dose (0.1 ml/10 g body wt) of physiological saline solution was given via the jugular catheter as soon as intravenous access was established followed by a continuous infusion of 0.2 ml·10 g^−1^·h^−1^. This infusate contained 146 mM Na^+^, 5 mM K^+^, 113 mM Cl^−^, 15 mM HCO_3_^−^, 0.25% (wt/vol) FITC-inulin, and 0.5% (wt/vol) *p*-aminohippuric acid sodium salt (PAH; Sigma) at pH 7.4. Mean arterial blood pressure was recorded from the carotid catheter in real time.

#### Infusion protocols.

**Fig. 1.**
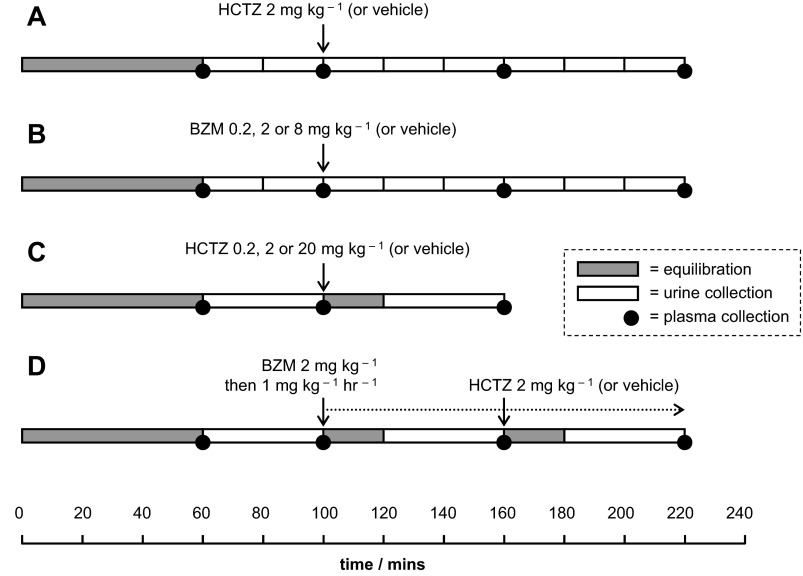
Renal clearance protocols. *A*: hydrochlorothiazide (HCTZ) time-course series. *B*: benzamil (BZM) time-course and dose-response series. *C*: HCTZ dose-response series. Some data for the 2 mg/kg group were derived from the relevant collections from the time-course series (*clearance protocol A*). *D*: protocol designed to assess the natriuretic interaction between BZM and HCTZ. All drugs were administered in 0.9% NaCl and 1% DMSO except for 20 mg/kg HCTZ, for which the concentration of DMSO was 2%. In the HCTZ dose-range experiment (*clearance protocol C*), two vehicle controls were used (containing 1 or 2% DMSO), but as there were no differences between these groups, their data were pooled for the final analysis.

Four different protocols of drug administration and sample collection were used (Fig. 1). Blood samples (∼80 μl) were obtained from the arterial line at the end of the first equilibration period and after each urine collection; these were used to determine plasma inulin concentration, PAH concentration, and osmolality. At the end of the protocol, a 1-ml sample of blood was obtained for the measurement of plasma Na^+^ concentration and plasma K^+^ concentration.

#### Analyses.

The FITC-inulin concentration in plasma and urine was determined by measuring fluorescence intensity at pH 7.4 relative to a nine-point standard curve in duplicate. The PAH concentration in plasma and urine was determined by a colorimetric method based on Smith's modification to the acidic diazotization and coupling method of Bratton-Marshall relative to an 11-point standard curve in duplicate (47). Na^+^ and K^+^ concentrations were measured by an ion-sensitive electrode using a calibrated Roche 9180 Electrolyte Analyzer; osmolality was determined by freezing point depression on a Vogel Osmometer OM 801.

#### Equations.

The following equations were used:

GFR = C_inulin_

RBF = (C_PAH_/0.9) × [1/(1 − hematocrit)]

FE_Na_ = [(U_Na_ × P_inulin_)/(P_Na_ × U_inulin_)] × 100

TTKG = (U_K_/P_K_)/(P_Osm_/U_Osm_)

E-C_Osm_ = (U_Na+K_ × V)/P_Na_

E-C_H_2_O_ = V − E-C_Osm_

T_H_2_O_^c^ = E-C_Osm_ − V

DT_HCTZ_ = U_HCTZ_(P_Osm_/U_Osm_) where GFR is glomerular filtration rate, C_*X*_ is the renal clearance of *X* in the plasma, RBF is renal blood flow, FE_Na_ is the fractional excretion of Na^+^, U_*X*_ is the concentration of *X* in the urine, P_*X*_ is the concentration of *X* in the plasma, TTKG is the transtubular K^+^ gradient, P_Osm_ is plasma osmolality, U_Osm_ is urine osmolality, E-C_Osm_ is electrolyte osmolar clearance, V is the urine flow rate, E-C_H_2_O_ is electrolyte-free water clearance, T_H_2_O_^c^ is free water reabsorption, and DT_HCTZ_ is the concentration of HCTZ in the distal tubule.

#### Quantifying HCTZ by liquid chromotography-tandem mass spectroscopy.

Urine samples (20 μl) were spiked with 50 ng trichlorothiazide (TCTZ), which was used as an internal standard to correct for variation in the efficiency of HCTZ extraction on a sample-by-sample basis, and then extracted into 10 volume ethylacetate by rolling for 30 min at room temperature. The organic phase was dried down under nitrogen gas at 40°C and then reconstituted in 100 μl of 5% acetonitrile and 95% of 0.5 mM ammonium acetate. Liquid-chromatography-tandem mass spectrometry (LC-MS/MS) was performed on an Aria CTC Turboflow autosampler and HPLC system (Thermo Fisher) used with a TSQ Quantum Discovery triple-quadropole mass spectrometer. Analytic chromatography was performed using a T3 Atlantis column (2.1 × 100 mm, 3 μm, Waters, Manchester, UK) with a flow rate of 0.3 ml/min at 40°C with a gradient ranging from 5% to 95% acetonitrile over 6 min and an aqueous component of 0.5 mM ammonium acetate. The mass spectrometer was operated in negative ion electrospray ionization mode. Source conditions were as follows: 3-kV spray voltage, 60/5 sheath and auxiliary nitrogen gas, 300°C capillary temperature, and 1.5-mTorr argon collision gas. Results were compared with a 10-point standard curve constructed in mouse urine.

#### Statistical analysis.

Except where stated otherwise, data are presented as means and 95% confidence intervals for the mean. Experimental groups were compared by Student's *t*-test or ANOVA with appropriate post hoc testing using GraphPad Prism (version 5.01).

## RESULTS

### 

#### Natriuretic response to HCTZ.

**Fig. 2.**
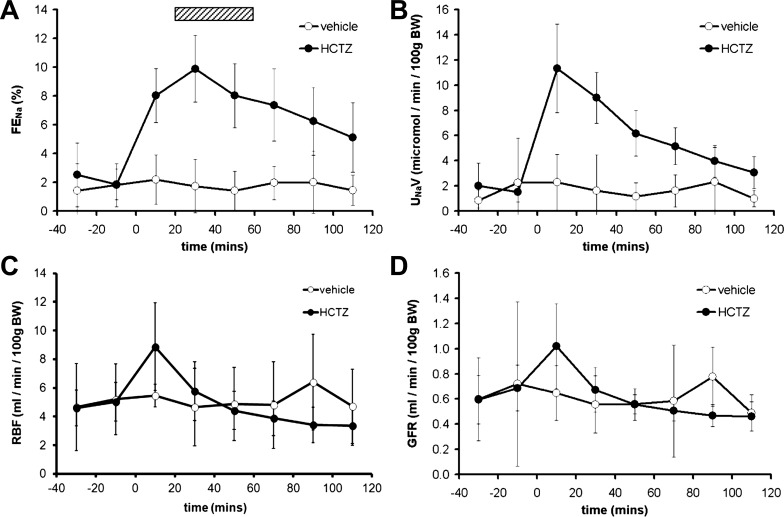
Natriuretic response to acute HCTZ. *A*: Time course of the acute natriuretic response to a bolus dose of HCTZ (2 mg/kg) expressed as the fractional excretion of Na^+^ (FE_Na_). *B*: data from the same experiment as in *A* presented as net urinary Na^+^ excretion (U_Na_V). *C*: renal blood flow (RBF) determined by *p*-aminohippuric acid sodium salt clearance. *D*: glomerular filtration rate (GFR) determined by inulin clearance. BW, body weight. Data are means ± 95% confidence intervals (CI).

Our first series of experiments established the time course for the renal response to an intravenous bolus of HCTZ (*clearance protocol A*; Fig. 1). An immediate increase in urinary Na^+^ excretion was observed, which peaked at 30 min and was sustained for at least 110 min (Fig. 2*A*). Although net urinary Na^+^ excretion was highest in the 20 min immediately after HCTZ administration (Fig. 2*B*), this peak coincided with peaks in RBF (Fig. 2*C*) and GFR (Fig. 2*D*) and was subject to anatomic and nonanatomic “dead space” artifacts. In subsequent experiments, we therefore allowed 20 min of reequilibration after drug administration before urine was collected over the following 40 min to capture peak natriuresis (hatched box in Fig. 2*A*).

**Fig. 3.**
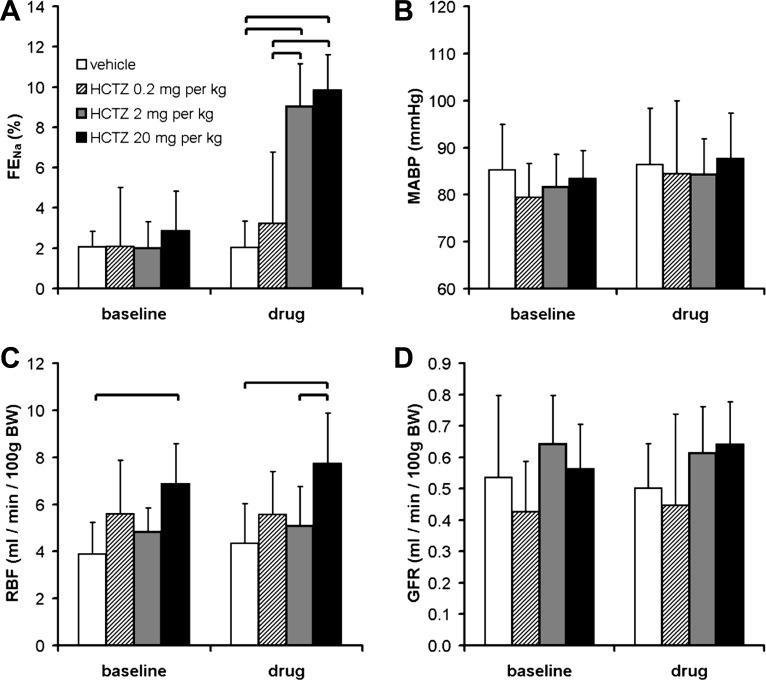
Dose-response relationships of HCTZ. *A*: effect of HCTZ dose on FE_Na_. This was assessed over a 40-min period after a bolus dose of drug, corresponding to the period delineated by the hatched box in Fig. 2*A*; see *clearance protocol C* in Fig. 1. *P* < 0.0001 for each of time, dose, and interaction by two-way ANOVA; the brackets indicate significant differences between treatment groups (*P* < 0.05 by post hoc Bonferroni's test). *B–D*: effect of HCTZ dose on mean arterial blood pressure (MABP; *B*), RBF (*C*), and GFR (*D*). The HCTZ dose had a significant effect on RBF (*P* < 0.01 by two-way repeated-measures ANOVA) but not MABP or GFR; the brackets indicate significant differences between groups (*P* < 0.05 by post hoc Bonferroni's test). Data are means ± 95% CI.

Similar renal clearance studies have used bolus doses of HCTZ ranging from 0.05 to 30 mg/kg (4, 5, 25). In our dose-range experiment (*clearance protocol C*; Fig. 1), HCTZ at 0.2 mg/kg did not elicit a significant increase in urinary Na^+^ excretion; doses of 2 and 20 mg/kg elicited natriuretic responses of equal magnitude (Fig. 3*A*).

**Fig. 4.**
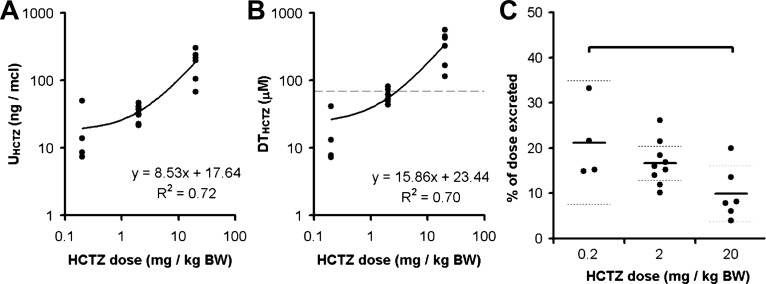
Concentration of HCTZ in the urine and tubular fluid. *A*: HCTZ concentration in the urine (U_HCTZ_) determined by liquid chromotography-tandem mass spectroscopy (LC-MS/MS). This measure varied in direct proportion to HCTZ dose. *B*: estimated concentration of HCTZ in the distal renal tubule (DT_HCTZ_). The horizontal dashed line represents the approximate IC_50_ for HCTZ in vivo (37). *C*: proportion of the total HCTZ dose that was excreted in the urine during the 40-min collection period. The thick and dotted horizontal lines mark the means and 95% CI, respectively. This measure was significantly lower in the 20 mg/kg BW group (*P* < 0.05 by one-way ANOVA and post hoc Bonferroni's test).

Urinary HCTZ excretion during the dose-range experiment was assessed by LC-MS/MS. As expected, the concentration of HCTZ in the urine varied in proportion to the administered dose (Fig. 4*A*). There was, however, a reduction in the proportion of the total dose that was excreted during the 40-min urine collection in mice that received 20 mg/kg, suggesting that the mechanisms mediating urinary HCTZ excretion became saturated at this higher dose (Fig. 4*C*). From in vitro studies of recombinant NCC (37) and micropuncture recordings of Cl^−^ concentration in the distal tubular fluid (26, 55), one can estimate that the IC_50_ for HCTZ at the NCC in vivo will be ∼70 μM. The estimated concentration of HCTZ in the distal tubular fluid (DT_HCTZ_) of mice that received a dose of 2 mg/kg clustered around this value, in keeping with this dose eliciting maximal natriuresis (Fig. 4*B*). We therefore selected a dose of 2 mg/kg for use in all subsequent experiments, minimizing any off-target effects in the proximal tubule. HCTZ can also affect blood pressure and renal hemodynamics (40), but no such changes were observed at this dose (Fig. 3).

#### Effect of HCTZ on renal water clearance.

**Fig. 5.**
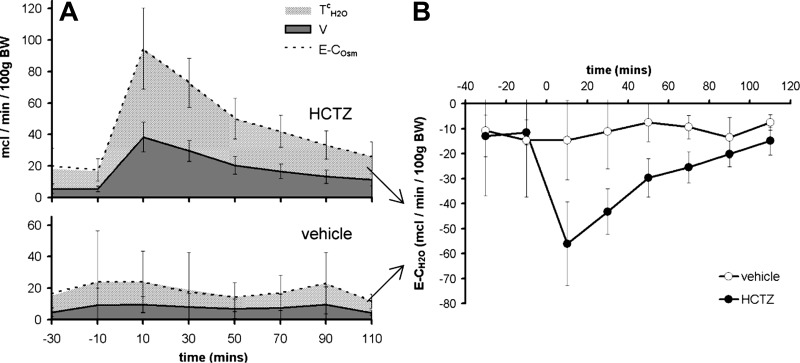
Effect of HCTZ on water metabolism. *A*: time course of changes in urine flow (V), electrolyte osmolar clearance (E-C_Osm_), and free water reabsorption (T_H_2_O_^c^) after HCTZ was determined using *clearance protocol A* (see Fig. 1). *B*: time course of electrolyte-free water clearance (E-C_H2O_), i.e., T_H_2_O_^c^ expressed as a negative value. Data are means ± 95% CI.

Mice exhibited negative free water clearance throughout the renal clearance protocol, whether this measure was calculated as electrolyte-free water (Fig. 5*A*) or as osmole-free water (data not shown). This perhaps reflects antidiuretic hormone production in response to anesthesia and surgery (6). As expected, HCTZ induced a further reduction in free water clearance (Fig. 5*B*), in keeping with the inhibition of Na^+^ reabsorption in the diluting segment (32, 50). As a consequence, HCTZ caused a reduction in plasma osmolality and plasma Na^+^ concentration (Table 2). Conversely, HCTZ caused a relative increase in hematocrit (Table 2).

**Table 2.** Biochemical parameters in the terminal blood sample

	*n*	P_Na_, mM	P_K_, mM	P_Osm_, mOsm	Hematocrit, %
HCTZ time course (*clearance protocol A*)					
Vehicle control	5	146.6 ± 2.6	4.1 ± 0.4	303.8 ± 3.8	37.4 ± 1.7
HCTZ (2 mg/kg)	9	143.1 ± 2.8	4.1 ± 0.2	296.8 ± 5.6	40.3 ± 2.6
HCTZ dose-range (*clearance protocol C*)					
Vehicle control	9	146.4 ± 1.3	4.5 ± 0.6	304.0 ± 4.0	40.4 ± 1.2
HCTZ (0.2 mg/kg)	4	145.8 ± 1.7	4.8 ± 0.6	306.3 ± 8.0	38.5 ± 4.7
HCTZ (2 mg/kg)	9	143.1 ± 2.8 *	4.1 ± 0.2	296.8 ± 5.6	40.3 ± 2.6
HCTZ (20 mg/kg)	8	143.6 ± 1.8	3.9 ± 0.3	296.9 ± 4.9	44.2 ± 1.3 *
HCTZ and BZM (*clearance protocol D*)					
BZM plus vehicle control	6	142.2 ± 2.2	5.8 ± 0.2	306.0 ± 3.0	38.3 ± 3.2
BZM plus HCTZ	6	139.7 ± 3.9	5.5 ± 0.4	301.5 ± 5.8	43.2 ± 2.5 *

Data are means ± 95% confidence intervals; *n*, no. of animals/group. P_Na_, concentration of Na^+^ in the plasma; P_K_, concentration of K^+^ in the plasma; P_Osm_, plasma osmolality.

* *P* < 0.05 for significant difference from the control group within each experiment by an unpaired *t*-test (*clearance protocols A* and *D*) or by a post hoc Dunnett's test after one-way ANOVA (*clearance protocol C*).

#### Natriuretic response to BZM.

**Fig. 6.**
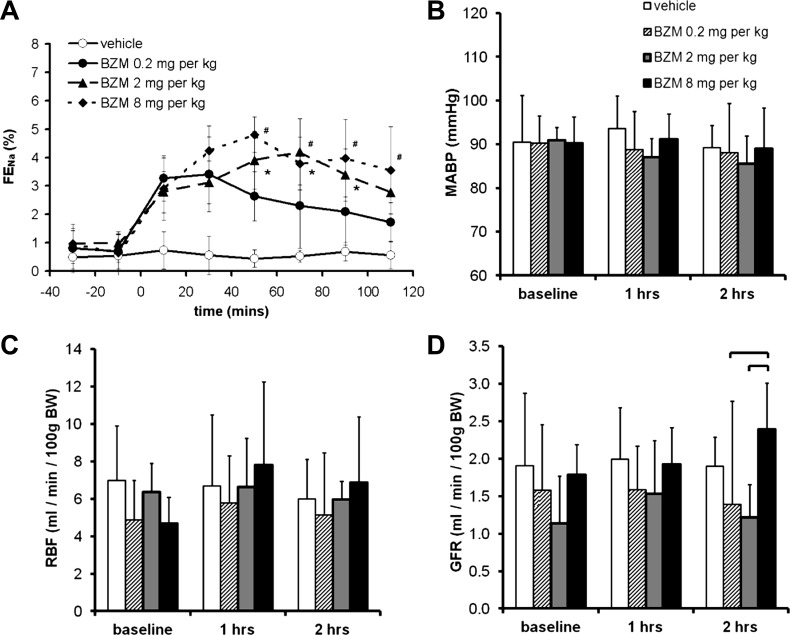
Dose-response relationships of BZM. *A*: effect of BZM dose on FE_Na_ determined using *clearance protocol B*. Both time and dose were significant sources of variation (*P* < 0.0001 by two-way ANOVA). *The 2 mg/kg group significantly different from the 0.2 mg/kg group; #the 8 mg/kg group significantly different from the 0.2 mg/kg group (*P* < 0.05 by post hoc Bonferroni test). The 2 and 8 mg/kg groups did not differ significantly at any time point. *B*: effect of BZM dose on MABP (there was no significant effect of time or dose by two-way ANOVA). *C*: effect of BZM dose on RBF (there was no significant effect of time or dose). *D*: effect of BZM dose on GFR (*P* < 0.05 for dose by two-way ANOVA; the brackets indicate significant differences between groups: *P* < 0.05 by post hoc Bonferroni test). Data are means ± 95% CI.

**Fig. 7.**
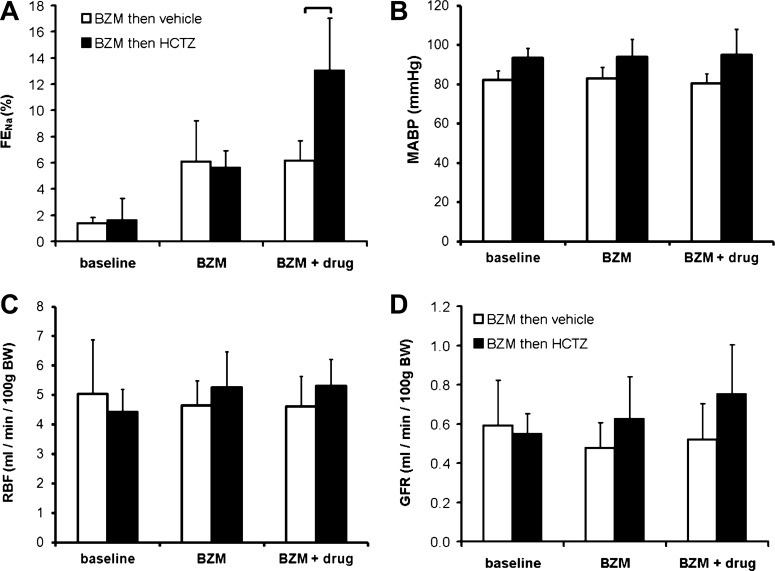
Interaction between HCTZ and BZM. The interaction between BZM and HCTZ was evaluated using *clearance protocol D* (Fig. 1). *A*: FE_Na_. The brackets indicate significant differences between groups (*P* < 0.05 by post hoc Bonferroni's test after two-way repeated-measures ANOVA). *B–D*: MABP (*B*), RBF (*C*) and GFR (*D*). Neither drug exerted a significant effect on MABP, RBF, or GFR (*P* > 0.05 for time, HCTZ, and interaction by two-way ANOVA). Data are means ± 95% CI.

We used BZM to inhibit ENaC, as this compound exhibits less off-target inhibition of Na^+^/H^+^ exchanger 2 than amiloride (22, 59). A bolus of BZM elicited a natriuresis that was sustained for at least 110 min. The magnitude of this response was larger in mice that received 2 mg/kg than in those that received 0.2 mg/kg, but no further increases were observed in mice that received 8 mg/kg (Fig. 6*A*). There was no significant hemodynamic effect of any of the three doses except for an apparent increase in GFR after 60 min with the highest dose (Fig. 6, *B–D*). Therefore, for our definitive experiment (*clearance protocol D*), we chose a bolus dose of 2 mg/kg BZM followed by a continuous infusion of 1 mg·kg^−1^·h^−1^. This regime induced maximal ENaC blockade that remained stable over the study period (Fig. 7*A*, vehicle-treated group), without affecting blood pressure, RBF, or GFR (Fig. 7, *B–D*).

#### Interaction between HCTZ and BZM.

In light of the results described above, we arrived at a renal clearance protocol capable of examining the acute response to NCC inhibition superimposed on constant ENaC blockade (*clearance protocol D*; Fig. 1). We used this to test our central hypothesis: that the natriuresis induced by HCTZ would be augmented by concomitant ENaC blockade. However, during BZM infusion, HCTZ elicited a natriuresis of a magnitude similar to that in diuretic-naïve mice (cf. Figs. 7*A* and 3*A*). An analysis of the HCTZ-induced increment in FE_Na_ (ΔFE_Na_) in individual BZM-treated and BZM-naïve mice confirmed that the response did not differ between these groups and, therefore, that the natriuretic effects of HCTZ and BZM were additive (see Fig. 9*C*). (The results were similar if expressed as change in net urinary Na^+^ excretion; data not shown.)

#### Kaliuretic response to HCTZ.

**Fig. 8.**
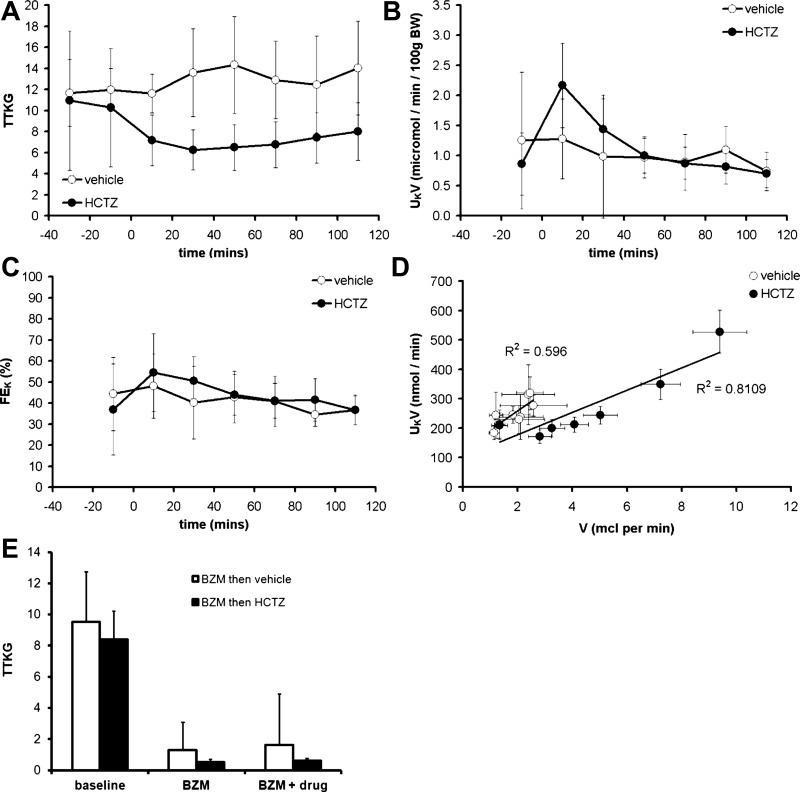
Kaliuretic effect of HCTZ. *A*: time course of the effect of HCTZ on the transtubular K^+^ gradient (TTKG) determined using *clearance protocol A* (see Fig. 1). *B*: time course of net urinary K^+^ excretion (U_K_V). *C*: time course of the effect on fractional K^+^ excretion (FE_K_). *D*: relationship between V and U_K_V; data were obtained from the time-course series with each point representing a different urine collection period. *E*: effect of BZM on TTKG determined using *clearance protocol D*. Data are means ± 95% CI.

**Fig. 9.**
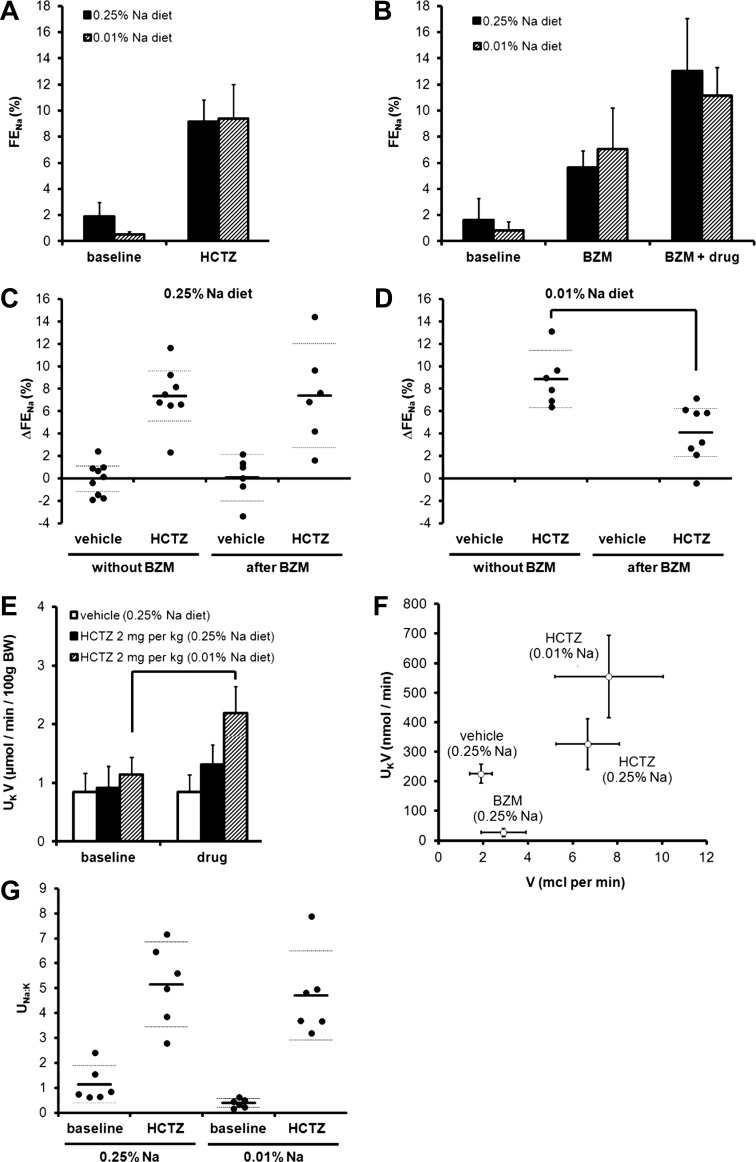
Interaction between HCTZ and BZM in mice adapted to dietary Na^+^ restriction. *A*: natriuretic response to an acute bolus of 2 mg/kg HCTZ (*clearance protocol C*) in mice after 3 days of the 0.01% Na^+^ diet. Data are presented alongside those obtained in mice on a control diet (i.e., the same data as shown in Fig. 3*A*). (There was no significant effect of diet by two-way ANOVA.) *B*: natriuretic response to HCTZ and BZM in mice after 3 days of the 0.01% Na^+^ diet determined using *clearance protocol D*. Data are presented alongside controls (i.e., as shown in Fig. 7*A*). *C* and *D*: data from the same experiments used to generate *A* and *B* plotted to show the vehicle- or HCTZ-induced increment in FE_Na_ (ΔFE_Na_) for each mouse. All data were derived from the final urine collection period. HCTZ-induced ΔFE_Na_ was no different in the presence of BZM on a control diet (no significant effect of BZM by two-way ANOVA) but was significantly lower in the presence of BZM after dietary Na restriction (*P* < 0.05 by *t*-test). *E*: U_K_V after a bolus dose of HCTZ (*clearance protocol C*). There was no increment in U_K_V after vehicle or after HCTZ in mice maintained on a control diet, but there was a significant increase after HCTZ in mice adapted to a low-Na^+^ diet (*P* < 0.0001 for time; *P* < 0.01 for experimental group; *P* < 0.01 for interaction by two-way ANOVA; *P* < 0.05 for the comparison between baseline and drug periods in the 0.01% Na^+^ group only by post hoc Bonferroni test). *F*: effect of HCTZ and BZM on the relationship between V and U_K_V. Each point represents the second period of urine collection in *clearance protocol C* (vehicle and HCTZ) or *clearance protocol D* (BZM). *G*: urinary Na^+^-to-K^+^ concentration ratio (U_Na:K_) after HCTZ (*clearance protocol C*). All data are means ± 95% CI.

HCTZ induced a sustained reduction in TTKG, indicative of suppressed K^+^ secretion in the cortical CD (Fig. 8*A*). Although there was a transient increase in net urinary K^+^ excretion immediately after HCTZ administration, we attribute this to a dead-space artifact (Fig. 8*B*); HCTZ did not affect fractional K^+^ excretion (Fig. 8*C*). Any effect of HCTZ on renal K^+^ excretion was dominated by its effects on the urine flow rate (Fig. 8*D*). In contrast, BZM induced a sharp reduction in TTKG and a dissociation between urinary flow rate and K^+^ excretion (Figs. 8*E* and 9*F*). HCTZ had no effect on plasma K^+^ concentration (Table 2).

#### Effect of dietary Na^+^ restriction.

Our failure to detect either a supra-additive interaction between HCTZ and BZM or an acute kaliuretic response to HCTZ alone might be due to low ENaC activity in mice maintained on a control (0.25% Na^+^) diet. Therefore, we repeated the experiments in mice adapted to dietary Na^+^ restriction (3 days of 0.01% Na^+^), a maneuver that suppressed baseline FE_Na_ (Fig. 9, *A* and *B*). There were trends toward increased natriuretic responses to HCTZ alone and BZM alone (Fig. 9, *A* and *B*), but these did not reach statistical significance (*P* > 0.05 by *t*-test to compare HCTZ-induced or BFZ-induced changes in FE_Na_ between 0.25% Na^+^- and 0.01% Na^+^-treated groups). The combined natriuretic effect of BZM and HCTZ in Na^+^-deprived mice was subadditive (Fig. 9*D*).

However, in contrast to the results obtained on the control diet, HCTZ induced an acute kaliuresis in Na^+^-deprived mice (Fig. 9*E*) and a leftward shift in the flow-kaliuresis relationship (Fig. 9*F*). The urinary Na^+^-to-K^+^ concentration ratio after HCTZ was similar in Na^+^-replete and Na^+^-deprived mice (Fig. 9*G*).

## DISCUSSION

We tested the hypothesis that acute inhibition of NCC transport activity would enhance ENaC-mediated Na^+^ reabsorption and thereby induce kaliuresis. Our results disprove this: the natriuretic effect of HCTZ was not enhanced during ENaC blockade and a bolus of HCTZ did not enhance urinary K^+^ excretion in mice maintained on a control diet.

### 

#### Optimized protocols for diuretic pharmacotyping by renal clearance.

Thiazides are commonly used to study NCC function in mice, but there is little agreement on the optimal dose or the best sampling strategy. In renal clearance studies, bolus doses of HCTZ have ranged from 0.05 to 30 mg/kg and urine samples have been obtained immediately after drug administration for a period of up to 120 min in anesthetized mice or up to 6 h in conscious mice (4, 5, 25). The validity of such a diuretic pharmacotyping approach is critically dependent on the experimental design: the diuretics and their doses should be selected so that they exert specific inhibition of the solute transporter in question without perturbing hemodynamic parameters or exerting off-target effects on solute transport elsewhere in the renal tubule. Furthermore, urine should be collected during a period that best represents the natriuretic response, without the confounding influence of dead space effects. We therefore aimed to optimize protocols to aid future pharmacotyping studies. We bolstered our renal clearance experiments with an analysis of HCTZ concentration in the urine. DT_HCTZ_ was estimated in an analogous fashion to TTKG, by correcting the urinary HCTZ concentration for water reabsorption in the CDs, so that DT_HCTZ_ = U_HCTZ_ (P_Osm_/U_Osm_). This measure will therefore overestimate the HCTZ concentration in the tubular fluid at the site of pharmacological activity, the DCT, where tubular fluid is hypotonic with respect to plasma (21). Nevertheless, DT_HCTZ_ should provide useful information regarding the approximate concentration of HCTZ at the site of its pharmacological activity.

HCTZ and BZM may exert off-target effects on vascular function, either directly or indirectly through connecting tubule glomerular feedback (cTGF), whereby Na^+^ reabsorption in the CNT causes afferent arteriolar dilatation via an ENaC-dependent pathway (54). We detected an increase in RBF after high doses of HCTZ (20 mg/kg), an effect compatible with cTGF. However, this was more likely to have arisen from the direct vasodilator effects of HCTZ (40); we did not find any other effects of HCTZ or BZM on RBF or GFR in a direction compatible with the expected modulation of cTGF. Although our results suggest that NCC inhibition does not cause an immediate increase in Na^+^ flux through ENaC, they do not necessarily contradict the observation that HCTZ augments cTGF in rats (54). The time course of the cTGF response to thiazides has yet to be elucidated. Furthermore, the channels that participate in cTGF are almost certainly a subset of the total population of ENaC present in the CNT and CD; therefore, the thresholds above which variation in Na^+^ delivery will have a discernible effect on cTGF or on K^+^ excretion may differ.

Our results were obtained under anesthesia, which does diminish the potential for their translation to conscious animals and humans. Anesthesia can exert cardiodepressant effects and perturb vascular tone, RBF, glomerular filtration, and tubular reabsorption (31, 41). However, we used a regime that exerts minimal effects on renal function in rodents (43) and that, for our purposes, provided stability in blood pressure, RBF, and GFR for the duration of each experiment (e.g., Fig. 7, *B–D*). Thiobutabarbital anesthesia and vascular cannulation are expected to inhibit proximal tubular reabsorption (17) and to stimulate the production of renin (34) and vasopressin (6). The net effect would be to preserve the delivery of NaCl to the distal tubule and to stimulate ENaC expression (49), both desirable phenomena in a study designed to evaluated the interaction between NCC and ENaC.

#### Additive natriuretic interaction between HCTZ and BZM.

Combinatorial HCTZ and BZM exerted additive natriuresis. These data agree with chronic balance studies in humans in which the natriuretic effects of HCTZ and triamterene or amiloride were additive (16, 28, 36). They also agree with the observation that in segments exhibiting both NCC and ENaC activity (e.g., the rabbit CNT), thiazides have no immediate effect on transepithelial voltage (45).

Our findings refute the hypothesis that acute inhibition of NCC results in an immediate increase in Na^+^ flux through ENaC. One possibility is that HCTZ does not increase acute Na^+^ flux through ENaC in our model because Na^+^ delivery to the CNT and CDs is such that ENaC is saturated in the basal (diuretic-naïve) state. Thus, inhibition of NCC can evoke no further effect on ENaC activity. Conversely, stimulation of NCC (as in Gordon's syndrome) might. In keeping with this, a mouse model of Gordon's syndrome exhibits hyperkalemia despite increased abundance and proteolytic cleavage of ENaC subunits, increased abundance of maxi-K subunits, and an increase in the lumen-negative amiloride-sensitive potential in microperfused cortical CDs (58). However, the natriuretic interaction between HCTZ and BZM was not supraadditive even after dietary Na^+^ restriction, a potent stimulus to ENaC expression.

We propose that inhibition of NCC results in increased electrogenic Na^+^ reabsorption only after a period molecular and/or structural adaptation. An analogous situation exists with respect to the natriuretic interaction exhibited by loop and thiazide diuretics in rats. The natriuretic effects of acute bendroflumethiazide and acute furosemide were additive, whereas the effects of acute bendroflumethiazide and chronic furosemide were supra-additive (20), implying that increased thiazide sensitivity in furosemide-treated rats did not arise from increased distal Na^+^ delivery per se but rather from molecular or structural adaptations in the DCT. This effect is likely to account for the supra-additive effects of loop and thiazide diuretics in humans (24, 30).

#### Acute effect of HCTZ on renal K^+^ excretion.

In keeping with the additive natriuretic interaction between HCTZ and BZM, HCTZ did not elicit sustained increases in urinary K^+^ excretion. K^+^ secretion in the distal nephron is mediated by ROMK and BK (maxi-K) channels, with the latter activated by high urinary flow rates (2, 39). In balance studies in humans, thiazides elicit a kaliuresis within 12 h (23, 42); the classical explanation of thiazide-induced hypokalemia invokes a contribution from increased ENaC activity, favoring K^+^ secretion through ROMK, and from flow-induced BK activation (9). Studies using in vitro micropuncture and microperfusion to determine the effect of HCTZ on K^+^ transport in the rat distal tubule have yielded conflicting results. In some studies (19, 57), HCTZ stimulated K^+^ secretion by the distal tubules/CDs; in another study (53), chlorothiazide had no effect on rates of K^+^ transport in distal tubules. However, due to their technical challenge, micropuncture studies are ill placed to assess the immediate effects of HCTZ, with electrolyte transport usually being evaluated over a period of 2–3 h after thiazide administration.

In our study, although urinary Na^+^ excretion remained elevated for at least 110 min after HCTZ administration, K^+^ excretion was not elevated over this period. There was a positive relationship between urinary flow and K^+^ excretion, but, if anything, the gradient of this slope was reduced after HCTZ (Fig. 8*D*), suggesting impaired K^+^ excretion.

After dietary Na^+^ restriction, HCTZ did induce an acute kaliuresis. This was probably not the result of increased Na^+^ delivery to ENaC in the CNT and CDs, because the natriuretic effects of HCTZ and BZM were not supra-additive even after Na^+^ restriction. It could plausibly arise from HCTZ-induced flow activation of BK channels. Some investigators have reported that aldosterone stimulates the expression of BK channel subunits in the apical membranes of β-intercalated cells (56), although dietary Na^+^ restriction was not found to increase flow-stimulated K^+^ secretion in rabbits (10). Alternatively, dietary Na^+^ restriction may have facilitated kaliuresis via an aldosterone-dependant stimulation of Na^+^-K^+^-ATPase activity in the CDs, favoring K^+^ transport across the basolateral cell membrane (15).

#### Perspective: implications for the regulation of renal K^+^ excretion.

Na^+^ and K^+^ homeostasis depends on the coordinated action of an array of pumps and transporters in the distal renal tubule. These are regulated by neurohormonal inputs, whose signals converge on intracellular signaling pathways, notably the WNK-SPAK system. Current models propose that WNK kinases act as a molecular “switch” capable of setting the balance between electroneutral Na^+^ reabsorption in the DCT and electrogenic Na^+^ reabsorption via ENaC in the CNT and CDs, providing a molecular solution to the aldosterone paradox (1, 18). This system is therefore able to induce coordinated changes in solute transporter expression and activity, and perhaps also in gross epithelial structure, in response to physiological perturbations.

The regulation of NCC activity plays a key role in the maintenance of K^+^ balance. Dietary K^+^ manipulation can modulate NCC activity in a way that would be expected to restore K^+^ homeostasis: K^+^ restriction increases the expression and phosphorylation of NCC, whereas K^+^ loading causes dephosphorylation (13, 51). Recent data have revealed that this latter response occurs rapidly in mice (48). An enteral K^+^ load increased urinary K^+^ excretion and NCC dephosphorylation within 15–30 min, with proteolytic activation of ENaC being detectable only after 6 h. This temporal separation may suggest that immediate kaliuretic effect of NCC inhibition is mediated solely by increased delivery of Na^+^ to ENaC-expressing segments (33). Our data do not fit with such a model. While there is clearly an association among enteral K^+^, NCC phosphorylation status, and renal K^+^ excretion, causality has yet to be established. Indeed, the K^+^-induced kaliuresis was no different in NCC knockout mice than in wild-type mice (48). Taken together, these studies provide an impetus for further research into the molecular mechanisms responsible for fine tuning solute transport in the distal renal tubule on a minute-to-minute basis.

#### Conclusions.

In summary, an acute bolus of HCTZ induced a natriuresis that was no greater during concomitant ENaC blockade and acute HCTZ did not increase urinary K^+^ excretion in mice maintained on a control diet. Our results support a model in which inhibition of NCC activity does not increase Na^+^ reabsorption through ENaC solely by increasing distal Na^+^ delivery but rather by inducing a molecular and structural adaptation in downstream nephron segments.

## GRANTS

R. W. Hunter received an Edinburgh Clinical Academic Track Lectureship from the Wellcome Trust, which funded this work. E. Craigie was also funded by the Wellcome Trust. The authors acknowledge the assistance of the British Heart Foundation Centre of Research Excellence Award and of Kidney Research UK.

## DISCLOSURES

No conflicts of interest, financial or otherwise, are declared by the author(s).

## AUTHOR CONTRIBUTIONS

Author contributions: R.W.H., J.J.M., and M.A.B. conception and design of research; R.W.H., E.C., N.Z.M.H., and M.A.B. performed experiments; R.W.H. analyzed data; R.W.H. and M.A.B. interpreted results of experiments; R.W.H. prepared figures; R.W.H. drafted manuscript; R.W.H., E.C., and M.A.B. edited and revised manuscript; R.W.H., J.J.M., and M.A.B. approved final version of manuscript.
